# Evaluation of Blood Titanium Levels and Total Bone Contact Area of Dental Implants

**DOI:** 10.1155/2018/4121639

**Published:** 2018-06-26

**Authors:** Mustafa Temiz, Ertunc Dayi, Nesrin Saruhan

**Affiliations:** ^1^Department of Oral and Maxillofacial Surgery, Medipol University Faculty of Dentistry, Istanbul, 34214, Turkey; ^2^Department of Oral and Maxillofacial Surgery, Atatürk University Faculty of Dentistry, Erzurum, 25240, Turkey; ^3^Department of Oral and Maxillofacial Surgery, Eskisehir Osmangazi University Faculty of Dentistry, Eskisehir, 26000, Turkey

## Abstract

The purpose of this study was to evaluate the effect of total implant-bone surface contact area of dental implants applied on partial or total edentulous patients on the increase in the level of blood titanium level. Changes of the blood titanium levels were evaluated after placement of the dental implants in 30 patients including 15 females and 15 males. Patients were divided into 3 groups as dental implants were applied on only maxilla, only mandible, or both of them. Taking into the consideration anatomic formation and prosthetic indication, dental implant-bone total contact area was calculated and saved for each patient after dental implants placement. Blood samples of the patients taken preoperatively and postoperatively at 12 weeks were analyzed by ICP-MS device. Blood titanium levels of preoperative and postoperative blood samples were analyzed for each patient and results were evaluated statistically. In the evaluation after analyzing blood titanium level changes, while a statistically significant decrease was observed in Group 1 patients, a statistically significant increase was observed in Group 2 and Group 3 patients to blood titanium level. A statistically significant difference was observed between Group 1 and Group 2 and between Group 1 and Group 3 patients of blood titanium levels. The change of the blood titanium level was not related to total implant-bone surface area, number of the implants, and gender. In our study, no correlation was found between change of blood titanium level and total contact area with bone of dental implants. We believe that more accurate results can be obtained with biopsy of tissues and organs on animal studies.

## 1. Introduction

Although dental implants have been applied to replace lost natural dentition in order to regain chewing function, in recent years, expectations of individuals necessitate aesthetic placements as well [1]. Implants first appeared in the 1960s when Branemark, a Swedish scientist, described osseointegration, and this has been followed by the development of numerous implant systems. Today, researchers concentrate on various implementations in order to accelerate osseointegration of dental implants and increase implant-bone contact surface [2]. For this purpose, osseointegration surface areas are increased by roughening the surfaces of dental implant materials [3].

In modern implantology, pure titanium or titanium alloy dental implants are used. Characteristics of dental implants and their interactions with biological tissues are defined by means of titanium dioxide (TiO_2_) layer [4]. It has been reported that titanium and titanium alloys still undergo corrosion despite their significant resistance to corrosion due to the stability of TiO_2_ layer. Once the resistant oxide layer is disrupted or separated from part of the surface, titanium may corrode as much as other metals or may be eroded by the forces placed on it, and the oxide layer forming on it may be disintegrated [5]. Routine and widespread use of titanium-based implants has necessitated the control of corrosion products. The aim of this study is to investigate the effect of total titanium-bone contact area on the increase in blood titanium level. The number, diameter, and length of implants were taken into consideration when calculating implant's total titanium-bone contact area.

## 2. Materials and Methods

Patients in whom dental implant placement was planned following their radiologic and intraoral clinical examinations at the Department of Oral and Maxillofacial Surgery of Atatürk University Faculty of Dentistry were included in the study (Table 1).

After confirming that the patients meet the criteria specified in Table 1, informed written consent was obtained from each patient before they were included in the study. Implants with complications such as maxillary sinus or nasal mucosa perforation, bone dehiscence, bone defect, inadequate primary stability, and insufficient burial of implant were excluded from the study because of their possible negative effect on the study. Before starting this study planned on volunteer participants, ethical permission was obtained (Atatürk University Faculty of Dentistry Ethical Committee Decision No. 9/2013).

To form the patient groups, 10 patients were selected from the patients that have at least two indications for implant placement in their mandible, maxilla, or both mandible and maxilla. A total of 30 patients (15 females and 15 males) were included in the study (Table 2).

Implant Direct Legacy 3 (Implant Direct®, California, USA) implants were used in our study. Implants were applied to the mandible only, the maxilla only, or both the mandible and the maxilla during the same session, where none of the jaws required any augmentation. Giving due consideration to the manufacturer's diameters and lengths, implant diameters and lengths were planned according to the alveolar crest width and the distance of the alveolar crest tip to the anatomical structures.

**Figure 1:**
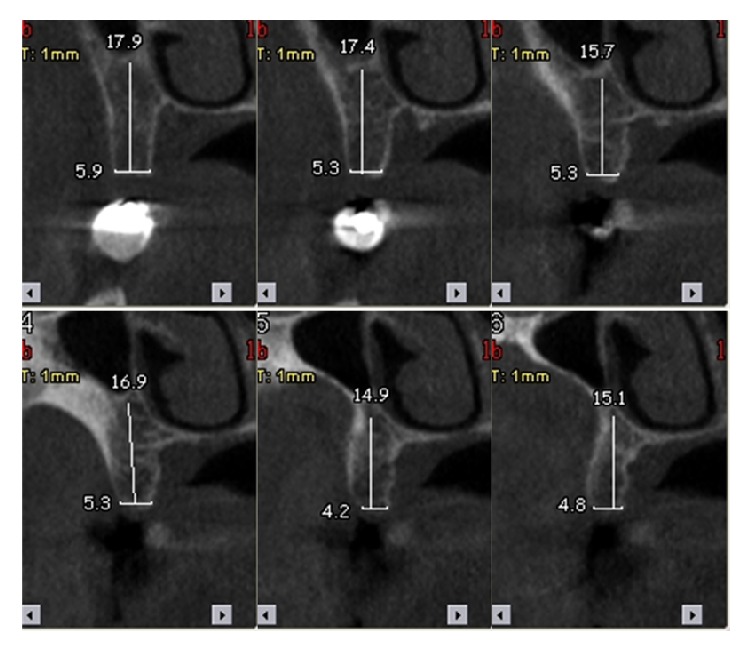
CBCT measurements.

In order to determine the implant length, cone beam computed tomography (CBCT) was used. Measurements were done on the obtained CBCT images (Figure 1). Based on the CBCT length measurements, the implants were applied at 8 mm, 10 mm, 11.5 mm, and 13 mm lengths.

Alveolar bone thickness was measured with CBCT to determine the implant diameter. Considering alveolar bone thickness and aesthetic criteria, implants with a diameter of 3.2 mm, 3.7 mm, 4.7 mm, and 5.7 mm were applied in accordance with mesial-distal, labiolingual thickness of alveolar bone and aesthetic criteria.

**Figure 2:**
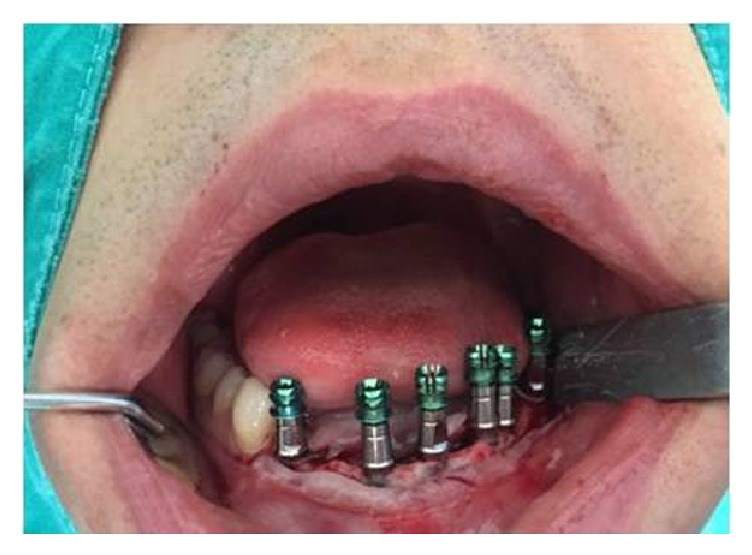
Placement of the implants at bone level.

Prior to implant placement, blood was drawn from the patients using 20 cc syringes (Hayat Sırınga, Istanbul, Turkey) and injected into 9 cc dry additive-free collection tubes (Vacutest Kima Srl, Piove di Sacco, Italy). Blood samples in tubes were stored at +4°C in a refrigerator. The patients were prepared according to the sterilization guidelines and local anesthesia was performed. To establish sterile surgical field, the perioral region was cleaned with 10% povidone iodine antiseptic solution and the oral cavity was rinsed with 0.12% chlorhexidine gluconate solution for one minute. Local anesthesia was performed with a local anesthetic that contains 40 mg articaine hydrochloride and 0.012 mg epinephrine hydrochloride per milliliter (Ultracain® D-S Forte, Sanofi-Aventis Deutschland GmbH, Germany). Prior to surgery, blood pressure was measured in every patient to make sure that their diastolic and systolic blood pressure readings are below 90 mmHg and 140 mmHg, respectively. A crestal incision suitable for the planned implant area was made and a mucoperiosteal flap was raised. Next, the implant hole was prepared. The drill system set forth in Implant Direct Legacy 3 surgical protocol was used in the order of the width to be 2.3 mm, 2.8 mm, 3.4 mm, 3.8 mm, 4.4 mm, and 5.1 mm. Implant holes were prepared at 800 rpm under saline cooling with minimum trauma to the bone. Implant holes were assessed using parallel pins in terms of angular relationship between the vertical axis of implant and adjacent teeth or implants. The implants were placed in a manner such that their titanium surfaces were completely covered by bone (Figure 2).

**Figure 3:**
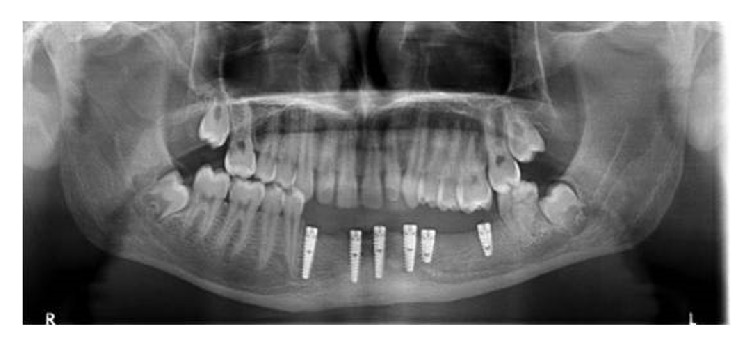
Postoperative panoramic radiography.

After placement of the implants in the bone, mucoperiosteal flaps were replaced and sutured with 3-0 silk. Postoperative control radiographs were taken (Figure 3). The patients that underwent implant surgery were prescribed an antibiotic (2 × 1) containing 875 mg amoxicillin and 125 mg clavulanic acid (Augmentin® BID tablet 1000 mg, GlaxoSmithKline, Brentford, United Kingdom), a nonsteroidal anti-inflammatory analgesic (2 × 1) naproxen sodium (Apranax Fort® film tablet 550 mg, Abdi Ibrahim Ilaç A.Ş., Istanbul, Turkey), and an oral rinse containing benzydamine hydrochloride + chlorhexidine gluconate (Andorex® 200 ml gargara, Pharmactive, Istanbul, Turkey). Following wound healing, 7 to 10 days after the surgery, sutures were removed.

After the implants were applied, implant number, length, and diameter were recorded in each patient. Total titanium-bone contact area for each implant was calculated using the implant manufacturer's titanium surface area data (Table 3).

The sum of implants' surface areas defines the total surface area where implants make contact with bone. Surface area for each patient in the groups was calculated and these values were arranged into tables (Tables 4–6).

**Figure 4:**
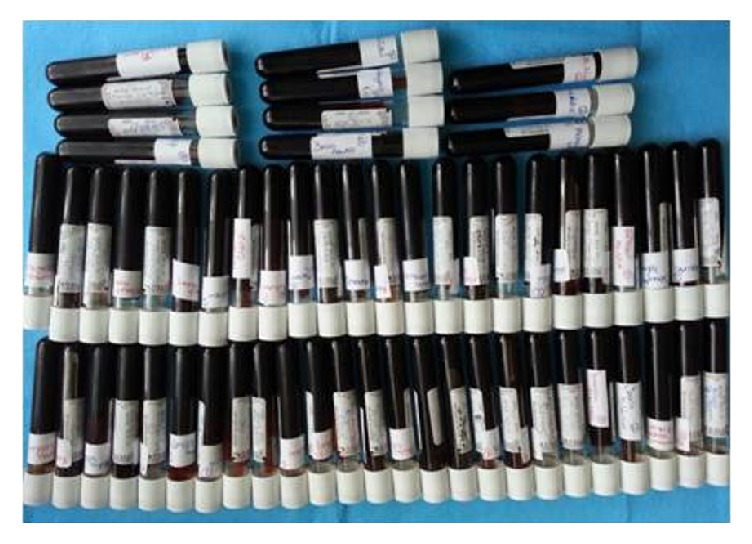
Blood samples from patients.

To determine preoperative blood titanium levels, blood samples were collected in additive-free tubes prior to surgery and stored at +4°C in a refrigerator. Second blood samples were collected 12 weeks postoperatively and stored in a refrigerator (Figure 4). Sixty tubes of blood samples were sent to Istanbul University Technology Transfer Placement and Research Center's Central Research Laboratory (MERLAB) to determine blood titanium levels.

Blood titanium levels were measured using Thermo Elemental X Series ICP-MS analyzer at MERLAB. Titanium levels of the blood samples were measured in mg/kg (ppm).

Statistical analyses were performed using SPSS v.16.0 (SPSS Inc., Chicago, IL, USA). Kolmogorov-Smirnov test was used to determine whether the sample data was normally distributed. To evaluate whether the change in blood titanium levels differed based on gender, the independent-samples *t*-test was used. One-way analysis of variance (ANOVA) test was used to investigate the change in blood titanium levels between the groups and Least Significant Difference (LSD) test was used to detect significant differences between the groups. Correlation analysis was used to evaluate whether implant surface area and implant number had a linear relationship with the change in blood titanium levels. Significance was set at *p* < 0.05.

## 3. Results

Our study included 15 (50%) females and 15 (50%) males with a mean age of 42.80 ± 12.04 years (range: 18 to 61 years). The number of implants applied to a single patient varied from 2 to 12.

Kolmogorov-Smirnov test showed that the change in blood titanium levels was distributed normally with respect to gender (*p* > 0.05). According to the independent-samples *t*-test results, there was a statistically significant difference (*p* < 0.05) in preoperative blood titanium levels between males and females. However, a significant difference in postoperative blood titanium levels between genders was not found (*p* > 0.05). In addition, there was no statistically significant difference between the changes in blood titanium levels for males and females (*p* > 0.05) (Table 7).

When the changes in blood titanium levels were compared between groups, Kolmogorov-Smirnov test result showed that the distribution of data was homogenous (*p* > 0.05); therefore, a one-way analysis of variance was performed (Table 8).

When the changes in blood titanium levels were compared between groups, a statistically significant difference was not found between the mandible only and the mandible + maxilla groups (*p* > 0.05). However, a significant difference existed between the maxilla only group and the other two groups (*p* < 0.05) (Table 9).

**Figure 5:**
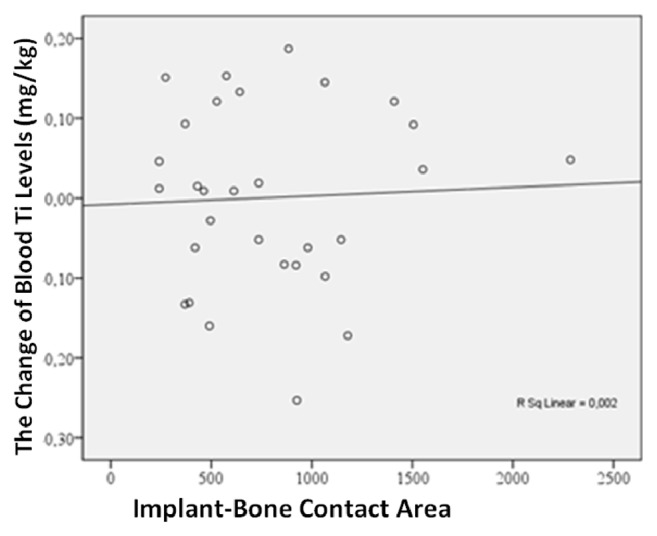
Correlation graph of change in implant-bone contact area and Ti level of blood.

**Figure 6:**
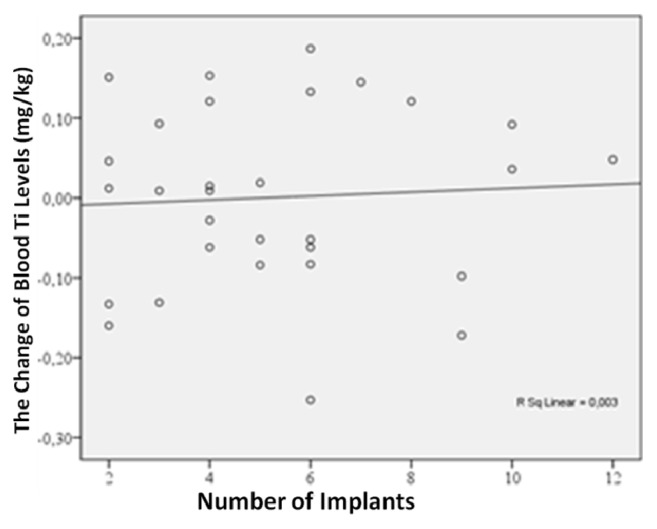
Correlation graph of change in blood level of Ti with implant number.

In order to determine the association of implant surface area and implant number with the change in blood titanium levels, a correlation analysis was performed and a significant association between implant surface area and the change in blood titanium levels was not found in any of the three groups (*p* > 0.05) (Figure 5). No significant correlation was found between implant number and the change in blood titanium levels (*p* > 0.05) (Figure 6).

## 4. Discussion

Implant-supported prosthetic placements are alternative treatment approaches that yield good results [6]. Titanium and/or titanium alloys (Ti6AL4V) are routinely used in healthcare industry for the manufacturing of various implants such as cervical prosthetics, joint prosthetics, mini-plates, mini-screws, and dental implants. Contemporary dental implants are screw-shaped titanium alloy structures that have a roughened surface and resemble a tooth root. Also, implant surfaces can be roughened by plasma spray, acid etching, and sandblasting. These processes are applied to the surface to increase implant-bone contact surface area, thus enhancing osseointegration potential of implants.

Frequent use of titanium-based materials and not knowing the systemic and local effects of titanium completely have concentrated studies on titanium. Because the volume and mass of titanium-based cervical, knee, and other total joint prosthetics are high, studies are mainly performed in orthopedics and toxicology. Titanium is widely used in food and paint industries as well. Because of its widespread use, concerns have been raised that titanium toxicity may have negative effects on human health such as inflammatory responses in lungs and malignancy [7, 8]. Epidemiological studies have shown that TiO_2_ neither has a carcinogenic effect on nor causes damage in the respiratory system [9]. The International Agency for Research on Cancer (IARC), however, has classified TiO2 as “possibly carcinogenic to humans” (Group 2B), based on inhalation studies that induced lung tumors in rats [10]. Oral, subcutaneous, and intraperitoneal administration did not increase the incidence of tumors in mice or rats. IARC has also evaluated implants of titanium or titanium-based alloys as “not classifiable as to their carcinogenicity to humans” (Group 3) [11].

In the study of Patton et al. [12] on orthopedic implants, an increase in serum titanium levels was observed; however, this increase was not found to be statistically significant. Karahalil et al. [13] conducted a genotoxicity study using micronucleus assay in patients who received mini-plates following orthognathic surgery or dental implants and collected oral mucosa samples from both patient groups. Analysis of samples revealed presence of titanium in oral mucosa in both groups; however, titanium levels were not high enough to cause DNA damage. In the study performed by Warheit et al. [14], titanium did not show a toxic effect on tissues. The authors stated that a high dose of 1000 mg/kg TiO_2_ did not cause any adverse effects in rats. They emphasized that researchers needed to conduct new studies to standardize information on titanium toxicity. The same authors also indicated that preterm birth, stillbirth, death, or toxicity symptoms were not seen when pregnant rats were given TiO_2_ nanoparticles [15]. Ipach et al. [16] investigated whether the number of screws and connectors used in spinal surgery had any correlation with blood titanium levels. They found no correlation between blood titanium levels and the number of titanium implants used for the surgery.

Wang et al. [17] detected TiO_2_ mainly in the liver, spleen, kidneys, and lungs of mice by ICP-MS. They identified liver damage, spotty necrosis of hepatocytes, and swelling of glomeruli. They reported an increase in ALT/AST ratio which is an indicator of liver damage.

Cundy et al. [18] studied whether an association existed between serum titanium levels and implant surface area following spinal fusion in children. The number of vertebral fusion levels, the number of pedicle screws, total rod length, and implant surface area were each found to have a significant association with postoperative serum titanium levels. In our study, the number of dental implants and implant-bone contact area did not have an association with the change in blood titanium levels. However, total surface area of dental implants is significantly smaller than that of orthopedic implants. Therefore, this relatively small surface area may have been the reason why the number of dental implants and total implant-bone contact area did not have a correlation with the change in blood titanium levels. It has been reported that corrosion products are carried through the bloodstream to distant sites such as hair, lungs, and spleen as their serum levels increase [19–21].

Although numerous studies on titanium toxicity exist, titanium's systemic effects and effects on individual organs, toxic dose range, and genotoxic activity have not been fully elucidated. In dentistry, titanium is used in the fabrication of numerous materials such as dental implants, mini-plates, mini-screws, endodontic files, and hand instruments. Despite its wide use in dentistry, very few studies about titanium have been done.

Baykus [22] investigated the effects of mini-plates and screws used in orthognathic surgery on the titanium levels of distant organs such as hair and nails. Samples were collected 14 to 96 months postoperatively. Analysis of samples revealed a significant increase in hair and nail titanium levels. Hair titanium levels in implant patients were 7 times higher than in control group. Nail titanium levels in implant patients were also significantly higher than in control group. The authors suggested that these significant increases in hair and nail titanium levels could have resulted from titanium-based implants placed in the patients.

Kılıc [23] studied whether serum titanium levels in dental implant patients caused an increase in interleukin-1 beta (IL-1*β*), interleukin-6 (IL-6), interleukin-10 (IL-10), and tumor necrosis factor alpha (TNF*α*). Regardless of the number, diameter, and length of the implants, two different brands of implants were used. Serum titanium and interleukin levels were measured preoperatively and 4 months postoperatively. The results of the study showed nonsignificant increases in IL-6, IL-10, and TNF*α* levels. However, a significant increase in IL-1*β* level was observed. Dental implants resulted in a nonsignificant increase in serum titanium level in all of the patients. In our study, an increase in serum titanium level was not seen in every patient who received dental implants. The serum titanium levels decreased in Group 1, whereas the levels increased in Groups 2 and 3; however, this increase was not statistically significant. We believe the different density and vascularization of upper and lower jaws may have affected the presence of a significant difference between Group 1 and the other two groups. The resistance of the alveolar bone to implant placement may result in small titanium pieces breaking off of implant surface, thus increasing corrosion. We think because the alveolar bone density is lower in the maxilla than in the mandible, it is harder to break titanium pieces off of implant surface and titanium undergoes less corrosion due to reduced stress acting on dental implants. In addition, because the maxilla is more vascular than the mandible, corrosion products in the maxilla can enter the circulation faster and accumulate in distant organs in a shorter time, making blood titanium level measurements misleading. However, the different density and vascularization between the maxilla and mandible fall short of explaining the reduction in blood titanium level. Also, we found a decrease in blood titanium levels for implants only into the maxilla. We thought that this decrease could be related to food and pharmaceuticals with no definite cause. Because titanium is used in numerous industries such as food and pharmaceuticals, therefore associating nonsignificant increases and decreases in blood titanium levels solely with dental implants may be misleading. Titanium toxicity studies on blood, blood products, tissue, and organs following dental implant placement should incorporate measurements of other metals present in the composition of dental implants such as aluminum and vanadium. Detecting the changes in the levels of these metals and whether these changes correlate with the changes in titanium levels can facilitate identifying the source as dental implants or not.

There was a statistically significant difference (*p* < 0.05) in preoperative blood titanium levels between males and females. We thought that the difference between females and males was related to some conditions for numerous industries such as food and pharmaceuticals, cosmetics, and herpes simplex, cheilitis, anti-inflammatory, anti-acne, and oral mucosa medications that contain TiO2.

## 5. Conclusion

Our study has shown that the changes in blood titanium levels can readily be determined. We think that this situation should be supported by animal studies, considering that titanium accumulates in tissues and organs.

## Figures and Tables

**Table 1:** Patient selection criteria.

Inclusion criteria	Exclusion criteria
(i) Being older than 18 years (ii) Being partially or totally edentulous (iii) Patients that have at least two indications for implant placement	(i) Presence of ischemic heart disease,
	(ii) Patients with a history of previous unsuccessful implant application,
	(iii) Patients with existing implants,
	(iv) Patients on continuous drug use,
	(v) Diabetic patients,
	(vi) Patients with renal failure,
	(vii) Patients with liver failure,
	(viii) Alcohol or drug addiction,
	(ix) Patients undergoing chemotherapy and/or radiotherapy,
	(x) Having bad oral hygiene,
	(xi) Patients with bleeding disorders,
	(xii) Pregnancy

**Table 2:** Number of patients and implants according to groups.

GROUPS	REGION	PATIENT	IMPLANT
GROUP 1	MAXILLA	10	43
GROUP 2	MANDIBLE	10	35
GROUP 3	MAXILLA + MANDIBLE	10	81

Total		30	159

**Table 3:** The surface areas according to diameter and length of implants.

Implant diameter (mm)/Length (mm)	8 mm	10 mm	11,5 mm	13 mm
3.2 mm	83.82 mm^2^	105.02 mm^2^	120.84 mm^2^	136.61 mm^2^
3.7 mm	106.32 mm^2^	139.46 mm^2^	182.57 mm^2^	195.85 mm^2^
4.7 mm	131.04 mm^2^	187.11 mm^2^	206.35 mm^2^	245.76 mm^2^
5.7 mm	157.15 mm^2^	-	-	-

**Table 4:** The number of implants and sum of the surface areas applied to Group 1 patients.

Patient	Implants	Surface area (mm^2^)
1	6	863.46
2	6	980.47
3	5	736.3
4	5	921.93
5	2	491.52
6	6	926.5
7	4	420.08
8	2	273.22
9	5	736.4
10	2	369.68

**Table 5:** The number of implants and sum of the surface areas applied to Group 2 patients.

Patient	Implants	Surface area (mm^2^)
1	2	241.68
2	4	575.87
3	2	241.68
4	4	431.82
5	3	463.25
6	4	496.48
7	6	642.36
8	4	612.84
9	3	370.81
10	3	391.34

**Table 6:** The number of implants and sum of the surface areas applied to Group 3 patients.

Patient	Implants	Surface areas (mm^2^)
1	6	885.09
2	12	2286.82
3	6	1146.42
4	8	1410.51
5	10	1553.02
6	7	1065.4
7	4	528.19
8	9	1179.35
9	9	1066.92
10	10	1506.2

**Table 7:** Preoperative and postoperative blood levels of Ti (mg/kg) according to gender.

	Female (*n* = 15)	*p*	Male (*n* = 15)	*p*	*p*
Blood Ti level (mg/kg)					
Preoperative	0.7490 ± 0.0544	0.985	0.8062 ± 0.0572	0.980	0.009^*∗*^
Postoperative	0.7496 ± 0.0977		0.8069 ± 0.0851		0.098
Difference	0.0006 ± 0.1192		0.0007 ± 0.1087		0.997

^*∗*^Statistically significant difference.

**Table 8:** Comparison of changes in blood Ti levels between groups.

	Sum of squares	df	Square of the averages	*F*	*p*
Between groups	0.080	2	0.040	3.778	0.036^*∗*^
Inside groups	0.285	27	0.011		

Total	0.364	29			

^*∗*^Statistically significant difference.

**Table 9:** Mean (±SD) values of change in blood Ti levels between groups (mg/kg).

		95% Confidence Interval		
Groups	Average ± SS	Lower Limit	Upper Limit	LSD Groups
Maxilla	−0.0719 ± 0.1070	−0.1485	0.0047	a
Mandible	0.0311 ± 0.0821	−0.0276	0.0898	b
Maxilla + Mandible	0.0428 ± 0.1159	−0.0401	0.1257	b

## Data Availability

The data used to support the findings of this study are available from the corresponding author upon request.
